# Contact & connect—an intervention to reduce depression stigma and symptoms in construction workers: protocol for a randomised controlled trial

**DOI:** 10.1186/s12889-015-2394-x

**Published:** 2015-10-16

**Authors:** Allison Milner, Katrina Witt, Lewis Burnside, Caitlyn Wilson, Anthony D. LaMontagne

**Affiliations:** Population Health Strategic Research Centre, School of Health & Social Development, Deakin University, Burwood, VIC Australia; Melbourne School of Population and Global Health, University of Melbourne, Melbourne, VIC Australia; Incolink, 1 Pelham Street, Carlton, VIC Australia

**Keywords:** Depression stigma, Mental health, Wellbeing, Suicide, Construction, Randomised controlled trial

## Abstract

**Background:**

Males employed in the construction industry have high rates of suicide. Although reasons underpinning this risk are multifaceted, poor help-seeking and stigma are represent major contributors. Males in the construction industry are also exposed to other risk factors for mental ill health and suicide, including unemployment. Sigma-reducing interventions that are accessible and attractive to recently unemployed males in the construction industry could therefore improve help-seeking, and address depression and suicidal behaviour in this population.

**Methods/Design:**

Contact&Connect will use a parallel individual randomized design to evaluate the effectiveness of a multimedia-based intervention aimed at reducing stigma. The intervention consists of a package of 12 brief contact interventions (BCIs) delivered over a six month period. BCIs will direct participants to informational programs and microsites. Content will address three major themes: debunking depression myths and stereotypes, normalisation, and empowerment. Target enrolment is 630 (315 in each arm), each to be followed for 12 months. Eligible participants will be males, between 30 and 64 years, unemployed at the time of recruitment, registered with Incolink (a social welfare trustee company for unemployed members of the construction industry), and own a smart phone with enabled internet connectivity.

**Discussion:**

At present, there are no programs that have been shown to be effective in reducing stigma in the blue-collar male population. Contact&Connect promises to provide a tailored, efficient, and scalable approach to reducing stigma, depressive symptoms and suicidality among unemployed males.

**Trial registration:**

Australian New Zealand Clinical Trials Register ACTRN12615000792527  (date of registration: 30 July, 2015).

**Electronic supplementary material:**

The online version of this article (doi:10.1186/s12889-015-2394-x) contains supplementary material, which is available to authorized users.

## Background

Males in the construction industry are at greater risk of suicide than those in the general working population [1]. The reasons underpinning the elevated risk of suicide among males in the construction industry are multifaceted, but likely involve poor help-seeking and high stigma against mental health issues. Evidence suggests for example that help-seeking rates for common mental health problems, including depression, are consistently lower in males compared to females [2]. Internalised stigma, or self-stigma, [3] and stoicism [4] may represent further barriers to help-seeking in males.

Due to the cyclical nature of construction work, males employed in the construction industry are also more likely to experience periods of unemployment than those in other industries [5]. This represents a further risk factor, as research consistently demonstrates that unemployment is associated with an elevated risk of mental health problems [6] and suicidal behaviour [1]. The experience of unemployment, and subsequent loss of the work-related role, social support, and sense of time structure and activity provided by employment [7], may compound feelings of self-stigma and shame associated with a mental health problem, further contributing to suicide risk. To some extent, the experience of unemployment in the construction industry is normalized, with workers anticipating periods without work as they move from project to project [8].

To date, there have been a limited number of intervention studies addressing stigma, help-seeking and overall mental health of males in the construction industry who have recently experienced unemployment. In 2010, a meta-analysis assessed the effectiveness of interventions on work participation and mental distress for unemployed adults [9]. This review identified just six studies fitting inclusion criteria, none of which were conducted in Australia. One of the reasons for the lack of intervention studies in this area is likely connected to the fact that unemployed workers are difficult to identify and engage. Because the workplace is no longer available as an intervention setting for these individuals, recently unemployed workers no longer have regular face-to-face contact with supervisors and co-workers who could provide social support, identify potential mental health problems, and help with accessing professional help. Given the feasibility challenges of face-to-face interventions for this population, there is a need to address the mental health risks associated with unemployment through different mechanisms, such as online and electronic devices.

Online and electronic interventions have been shown to be effective in reducing stigmatising attitudes towards depression [10], knowledge about evidence based treatment [10], help-seeking from family and friends [11], and symptoms of depression and anxiety [12]. However, their effectiveness in addressing stigma and mental health among males in the construction industry is, as yet, unknown. To address this, we describe the design of Contact&Connect, a parallel group randomised controlled trial designed to assess the effectiveness of a multimedia package of informational and educational resources on mental health self-stigma and mental health literacy in males previously working in the construction industry in the state of Victoria, Australia.

## Methods

### Study design

Contact&Connect is an individual-level two-arm randomised controlled trial (Additioanl File 1). A total of 630 participants (315 in each arm) will be recruited. Simple randomisation using a random numbers table generated by computerised software (Stata for Windows, version 13 [13]) will be used to establish the randomisation sequence. Participants will be allocated to either the intervention or wait-list control group by an offsite researcher working independently from the research team. Owing to the nature of this intervention, neither the participants themselves nor the intervention team will be blind to allocation. However, outcome assessors will remain blind to treatment allocation through the use of unique study identifiers.

### Intervention development framework

An intervention mapping approach [14, 15] will be used to develop and refine the Contact&Connect intervention strategy. We will draw on past research conducted by our industry partner, Incolink, to inform the needs assessment phase of this project and will conduct a matrix evaluation to determine the likely outcomes for behavioural change analysis. Incolink is a state-wide social welfare trustee company that provides financial support for construction workers during the times they are without work. Incolink has the support of unions and employers in the industry, and is well recognised and trusted by workers.

Next, we plan to conduct a focus group session with 20 participants to generate program ideas and intervention materials to ensure these address the requirements for behavioural change as set out in the matrix evaluation. Together with the research team, this focus group will assess the adequacy of and solicit suggestions for optimising: 1) industry/male blue-collar worker relevance and framing; 2) preferred modality of BCIs; 3) BCI frequency and timing; 4) the attractiveness, clarity, and usefulness of the content. BCI design and content with then be refined using focus group findings.

We will then consult with our media partner, Publicity Works, to draft the themes, content, scope, and sequence of the BCIs, and to subject these materials to beta-phase testing with our sub-sample of 20 participants to ensure materials meet the needs and expectations of our participant group. The final two phases of the intervention mapping approach involve rolling the intervention out to our full participant group and undertaking process evaluations to determine efficacy and to review potential mechanisms of attitudinal and behavioural change. As part of these evaluations, we also plan on conducting further qualitative interviews with a random sub-sample of 20 participants to assess perceived usefulness of the intervention materials.

### Study objectives and outcomes

The primary objective of this trial is to evaluate the effectiveness of Contact&Connect, a package of multimedia-based brief contact interventions (BCIs), compared to wait-list, on attitudes and beliefs about depression, help-seeking inhibition, shame, self-blame and social inadequacy at post-intervention (six months) and at 12 months following enrolment. Secondary objectives include the evaluation of this package of interventions on suicidal ideation and depression symptomatology 12 months following enrolment.

Additionally, the degree to which changes in attitudes and beliefs about depression, help-seeking inhibition, and shame, self-blame or social inadequacy mediate changes in suicidal ideation or depression symptomatology will also be assessed.

### Participants

Males between the ages of 30 and 64 years who are registered with Incolink, and who own a smart phone with internet connectivity with adequate data download capacity will be invited to participate. Only unemployed persons contacting Incolink over the three months recruitment procedure will be considered.

Study exclusion criteria includes females, those younger than 30 years or older than 64 years, and those without a smart phone, internet connectivity, or adequate data download capacity, and those with inadequate English language skills.

Written informed consent will be sought from all participants prior to allocation. Ongoing consent will be inferred from participants’ response or non-response to the intervention materials and follow-up questionnaires.

Participants will be allocated to either the intervention or control group by an offsite researcher working independently from the research team.

### Intervention condition

Participants assigned to the intervention arm of this trial will receive a series of 12 informational and educational BCIs delivered to their smart phone over an intervention period of six months. Specific BCIs will target the specific outcomes of the project. Thus, separate BCIs will target knowledge about risk and protective factors for depression and suicide, the importance of social support through connections with friends, family, work colleagues, dispelling myths about mental health problems (in others and the self), and improving knowledge about help-seeking and communication.

Contact&Connect will be delivered to participants’ smart phones via rich text messages with embedded hyperlinks to microsites and other digital resources such as videos and digital wallet cards. These additional resources will provide additional information on stigma, mental health and help seeking, provide links to sources of help whilst encouraging the establishment and maintenance of long-term contact with others. BCIs will be delivered using Whispir^TM^ enterprise edition; an automated and programmable SMS message and communication management system. In addition to allowing multiple types of communication at once, this software will enable us to track participants’ engagement with various BCI materials.

### Control condition

Participants randomised to the control condition will be allocated to a wait-list and will receive the intervention materials in full at the conclusion of the intervention period.

### Data collection

All participants will complete a baseline electronic survey comprising information on sociodemographic information at the time of study enrolment, knowledge of depression symptoms, attitudes towards those with depression, and stigma, shame, self-blame or social inadequacy. Follow-up surveys will take place immediately post-intervention (six months), and then again at 12 months following enrolment to collect information on changes in knowledge of depression symptoms, attitudes towards those with depression, stigma, shame, self-blame, and social inadequacy and also to assess the impact of the intervention on suicidal ideation and depression symptomatology.

Primary outcome measures will include: scores on the attitudes, beliefs, and help-seeking inhibition subscales of the Depression Stigma Scale (DSS; [10]) and the shame, self-blame and social inadequacy subscales of the Self-Stigma of Depression Scale (SSDS; [16]), and levels of perceived social support from family and friends using the Multidimensional Scale of Perceived Social Support (MSPSS; [17]). Secondary outcomes will include: suicidal ideation as measured by scores on the suicidality subscale of the General Health Questionnarire-28 (GHQ-28; [18]) and depression symptomatology as measured by scores on the Center for Epidemiologic Studies Depression scale-Revised (CESD-R; [19]) and the depression subscale of the Brief Symptom Inventory (BSI; [20]).

### Same size and power calculations

Assuming, in line with previous work on interventions to improve mental health literacy in other occupational groups [21], a difference of 0.73 in beliefs about depression treatment between the intervention and control groups at post-intervention, a minimum of 226 participants per group will be required to detect this difference at the nominal 0.05 significance level with 90 % power. For our secondary outcomes, our previous study has shown that 13 % of the working population report suicide ideation [22]. We believe it is realistic to expect a change from 13 % to 8 % prevalence following the trial. Power calculations indicate we will need 267 persons per group to detect this difference at the nominal 0.05 significance level with 80 % power. Assuming 30 % drop-out, we therefore aim to recruit a total of 630 newly unemployed participants over a three month period. This is a feasible and achievable goal, given that approximately 20 unemployed workers lodge a claim each day with Incolink. We will recruit over a period of three months, thus allowing us to obtain a sample size adequately powered to assess both primary and secondary outcomes.

### Statistical methods

Intention-to-treat analyses are planned to compare the effectiveness of the Contact&Connect package of interventions on post-intervention. To account for the presence of missing data at the post-intervention and follow-up assessments, we plan to utilise imputation from the participants’ own responses either prior to and, if available, subsequent to drop-out to estimate likely values for missing variables. Simulation work suggests these two methods demonstrate greatest accuracy, least bias, and least reduction in dispersion as compared to other imputation methods [23].

The efficacy of this intervention on both the primary and secondary outcomes will be assessed using multivariate regression modelling. As we anticipate that some participants may become re-employed during the intervention or follow-up period, analyses will be adjusted for number of days reemployed during the intervention and follow-up period. Analyses will be undertaken in Stata for Windows, version 13 [13].

The intervention will also be evaluated qualitatively *post hoc* for appropriateness, ability to engage participants, and overall impact using a semi-structured interview with a random sub-sample of 20 participants. Interviews will be conducted over the phone within eight weeks of the conclusion of the intervention by trained researchers. Data from these interviews will be analysed thematically using NVivo for Windows, version 10 [24].

## Discussion

The Contact&Connect intervention will design, implement and evaluate a multimedia package addressing depression self-stigma and mental health literacy in recently unemployed males previously working in the construction industry in Victoria, Australia. The intervention will be designed drawing on previous online interventions found to be effective in increasing help-seeking behaviour for mental health problems [10–12, 25], as well as those that have reduced suicidal behaviour in clinical populations [13]. The goal of this trial is to assess the effectiveness of this intervention in terms of both depression self-stigma, depression symptomatology, and suicidal behaviour, and also to ensure optimal tailoring and relevance through the use of qualitative interviewing techniques.

Our study subjects are blue collar males, a demographic that is acknowledged as particularly at risk of suicide [26] and yet less likely to seek help for mental health problems [27, 28]. One of the reasons for this may be due to the unsuitability of mainstream clinically-orientated treatment approaches to depression. To be effective for males, mental health programs should emphasise help-seeking as a responsible, independent and “male” way of taking action, and should position mental health problems as needing to be tackled proactively rather than experienced passively [28]. Developed male-centered programs need to highlight the importance of timeliness in regard to the escalation of mental health problems and specifically to seek help for symptoms before they begin to impinge on traditional masculine roles (e.g., breadwinner, father, husband, friend, worker) or result in suicidal thoughts or behaviours. These suggestions also align with other studies [27, 29], which have additionally suggested that males need feel that they are in control of their treatment; or at least part of a collaboration together with treatment providers. Last, to be effective, treatment and help must be perceived as genuine.

We seek to address these factors in Contact&Connect by developing the content and appearance of the program from the “ground up” in collaboration with focus groups composed of industry specialists and construction workers with a lived experience of mental health problems. This not only ensures that the program is relevant and tailored to the specific needs of participants, it also models a partnership approach to the development of solutions for mental health problems among males in the building industry. We seek to provide an industry-embedded program, thus facilitating uptake and longer-term sustainability of the program.

The study design has a number of strengths that will ensure scalability. First, a smart phone-based intervention is particularly suited to construction workers, given that over 90 % of employees in this industry own a smart phone. Second, working with a state-wide redundancy fund that has general acceptance in the industry and state-wide coverage means that we will able to obtain a large sample size to ensure adequate representativeness. Our partnership approach will also optimise “buy in” into the program and a greater sense of ownership, both of which are important when considering recruitment into the trial, retention of participants in the program, and its longer term sustainability.

Once established, Contact&Connect promises to be a cost-efficient prevention initiative that could be rolled out to a large number of people at the same time. Further, Contact&Connect could operate alongside other treatment or health promotion activities, and presents minimal risk to participants. If shown to be effective, Contact&Connect is likely to lead to a number of individual benefits (e.g., reduced stigma, improved mental health literacy, reduced psychological distress and suicidality) and wider societal benefits (e.g., improved health outcomes and greater economic productivity).

Limitations of this design include the potential confounding influence of reemployment. As reemployment is associated with improvements across a number of health domains, including reductions in mental ill health and depression symptomatology [30], the inclusion of individuals who were reemployed during the study may dilute any effect of the intervention package. However, it would be clearly inappropriate and unethical to request that participants refuse offers of employment during the 12 month intervention and follow-up period. Additionally, as the exclusion of these participants would have serious implications for statistical power, we decided to retain these individuals in all of our analyses. However, our analytic strategy will account for re-employment during the six month intervention period.

## Conclusion

Unemployment is a risk factor for the development of depression [6] and suicidal behaviour [1], particularly among males [31]. Given that poorer mental health literacy [32], self-stigma [3], and stoicism [4] represent major barriers to help-seeking for mental health problems among males the Contact&Connect intervention aims to improve mental health literacy and reduce stigma amongst unemployed males in the construction industry in an accessible and attractive manner to improve help-seeking behaviour. It is anticipated that these effects will also mediate reductions in depression symptomatology and suicidal ideation in this vulnerable population.

### Ethical considerations and study guidance

This study has received ethical approval from the Deakin University Human Research Ethics Committee (DUHREC) (Additional file 2: approval reference number: 2015-194).

### Trial status

This trial is currently recruiting participants for the focus group sessions, and will commence full enrolment in late January, 2016.

### Availability of data and materials

There is no data available as yet from this trial. At the conclusion of the trial, we are happy to collaborate with researchers who would like to use our data, providing they contact us and provide a written request, analysis plan and likely outcomes of their proposed analysis.

## Additional files

Additional file 1:
**CONSORT 2010 checklist.** (PDF 124 kb) Additional file 2:
**Ethics approval 2015-194.** (PDF 40 kb)

## References

[1] Milner A, Morrell S, LaMontagne A (2014). Economically inactive, unemployed and employed suicides in Australia by age and sex over a 10-year period: What was the impact of the 2007 economic recession?. Int J Epidemiol.

[2] Oliver M, Pearson N, Coe N, Gunnell D (2005). Help-seeking behaviour in men and women with common mental health problems: cross-sectional study. Br J Psychiatry.

[3] Clement S, Schauman O, Graham T, Maggioni F, Evans-Lacko S, Bezborodovs N (2015). What is the impact of mental health-related stigma on help-seeking? A systematic review of quantitative and qualitative studies. Psychol Med.

[4] Judd F, Komiti A, Jackson H (2008). How does being female assist help-seeking for mental health problems?. Aust N Z J Psychiatry.

[5] Labour Force Survey, Australia, Quarterly [category number: 6291.0.55.003]. Canberra, ACT: Australian Bureau of Statistics, 2015.

[6] McKee-Ryan F, Song Z, Wanberg C, Kinicki A (2005). Psychological and physical well-being during unemployment: a meta-analytic study. J Appl Psychol.

[7] Jahoda M (1981). Work, employment, and unemployment: Values, theories, and approaches in social research. Am Psychologist.

[8] ILO. The construction industry in the twenty-first century: Its image, employment prospects and skill requirements. Geneva, Switzerland: International Labour Office, 2001.

[9] Audhoe S, Hoving J, Sluiter J, Frings-Dresen M (2010). Vocational interventions for unemployed: Effects on work participation and mental distress: A systematic review. J Occupat Rehab.

[10] Griffiths K, Christensen H, Jorm A, Evans K, Groves C (2004). Effect of web-based depression literacy and cognitive-behavioural therapy interventions on stigmatising attitudes to depression. Br J Psychiatry.

[11] Christensen H, Griffiths K, Jorm A (2004). Delivering interventions for depression by using the internet: randomised controlled trial. BMJ.

[12] Proudfoot J, Clarke J, Birch M-R, Whitton A, Parker G, Manicavasagar V (2013). Impact of a mobile phone and web program on symptom and functional outcomes for people with mild-to-moderate depression, anxiety and stress: a randomised controlled trial. BMC Psychiatry.

[13] Luxton D, June J, Comtois K (2013). Can postdischarge follow-up contacts prevent suicide and suicidal behavior? A review of the evidence. Crisis.

[14] Bartholomew L, Parcel G, Kok G, Gottlieb N, Fernández M (2011). Planning Health Promotion Programs: An Intervention Mapping Approach.

[15] Bartholomew L, Parcel G, Kok G (1998). Intervention mapping: a process for designing theory- and evidence-based health education programs. Health Educat Behav.

[16] Barney L, Griffiths K, Christensen H, Jorm A (2010). The Self-Stigma of Depression Scale (SSDS): Development and psychiatric evaluation of a new instrument. Int J Methods Psychiatr Res.

[17] Zimet G, Dahlem N, Zimet S, Farley G (1988). The multidimesional scale of perceived social support. J Pers Assess.

[18] Goldberg D, Hillier V (1979). A scaled version of the General Health Questionnaire. Psychol Med.

[19] Eaton W, Muntaner C, Smith C, Tien A, Ybarra M, Maruish M (2004). Center for epidemiologic studies depression scale: review and revision (CESD and CESD-R). The Use of Psychological Testing for Treatment Planning and Outcomes Assessment.

[20] Derogatis L, Melisaratos N (1983). The brief symptom inventory: an introductory report. Psychol Med.

[21] Jorm A, Kitchener B, Sawyer M, Scales H, Cvetkovski S (2010). Mental health first aid training for high school teachers: a cluster randomized trial. BMC Psychiatry.

[22] Milner A, Page K, LaMontagne A. Perception of mattering and suicide ideation in the Australian working population. Comm Ment Health [under review]. 10.1007/s10597-016-0002-x 26939798

[23] Engles J, Diehr P (2003). Imputation of missing longitudinal data: a comparison of methods. J Clin Epidemiol.

[24] QSR International. NVivo qualitative data analysis software, version 10. QSR International Pty Ltd; 2012.

[25] Christensen H, Leach L, Barney L, Mackinnon A, Griffiths K (2006). The effect of web based depression intervention of self-reported help-seeking: randomised controlled trial. BMC Psychiatry.

[26] Milner A, Niven H, LaMontagne A. Suicide by occupational skill level in the Australian constuction industry: Data from 2001 to 2010. Aust N Z J Pub Health. 2014;38(281-5). 10.1111/1753-6405.12205 24890487

[27] Johnson J, Oliffe J, Kelly M, Galdas P, Ogrodniczuk J (2012). Men’s discourses of help-seeking in the context of depression. Sociol Health Illness.

[28] Hoy S (2012). Beyond men behaving badly: a meta-ethnography of men’s perspectives on psychological distress and help-seeking. Int J Men’s Health.

[29] O'Brien R, Hunt K, Hart G (2005). ‘It’s caveman stuff, but that is to a centain extent how guys still operate’: Men’s accounts of masculinity and help-seeking. Soc Sci Med.

[30] Rueda S, Chambers L, Wilson M, Mustard C, Rourke S, Bayoumi A (2012). Association of returning to work with better health in working-aged adults: a systematic review. Am J Public Health.

[31] Morrell S, Taylor R, Quine S, Kerr C (1993). Suicide and unemploument in Australia, 1907-1990. Soc Sci Med.

[32] Cotton S, Wright A, Harris M, Jorm A, McGorry P (2006). Influence of gender on mental health literacy in young Australians. Aust N Z J Psychiatry.

