# Supplementary material for: Contact & connect—an intervention to reduce depression stigma and symptoms in construction workers: protocol for a randomised controlled trial
Source: BMC Public Health. 2015 Oct 16;15:1062. doi: 10.1186/s12889-015-2394-x (PMC4609134; doi:10.1186/s12889-015-2394-x)
Supplement: Additional file 2: — Ethics approval 2015-194. (PDF 40 kb) [file 12889_2015_2394_MOESM2_ESM.pdf]

Human Research Ethics

Deakin Research Integrity
Burwood Campus
Postal: 221 Burwood Highway
Burwood Victoria 3125 Australia
Telephone 03 9251 7123

research-ethics@deakin.edu.au

Memorandum

To: Dr Allison Milner
Population Health

B

cc:

From: Deakin University Human Research Ethics Committee (DUHREC)

Date: 02 September, 2015

Subject: 2015-194

Contact & Connect: A randomised trial of a multimedia-based mental health promotion program

Please quote this project number in all future communications

The application for this project was considered at the DU-HREC meeting held on 24/8/2015.

Approval has been given for Dr Allison Milner, Population Health, to undertake this project from 2/09/2015 to 2/09/2019.

The approval given by the Deakin University Human Research Ethics Committee is given only for the project and for the period as stated in the approval. It is your responsibility to contact the Human Research Ethics Unit immediately should any of the following occur:

- Serious or unexpected adverse effects on the participants
- Any proposed changes in the protocol, including extensions of time.
- Any events which might affect the continuing ethical acceptability of the project.
- The project is discontinued before the expected date of completion.
- Modifications are requested by other HRECs.

In addition you will be required to report on the progress of your project at least once every year and at the conclusion of the project. Failure to report as required will result in suspension of your approval to proceed with the project.

DUHREC may need to audit this project as part of the requirements for monitoring set out in the National Statement on Ethical Conduct in Human Research (2007).

Human Research Ethics Unit
research-ethics@deakin.edu.au
Telephone: 03 9251 7123
